# Identification of the notothenioid sister lineage illuminates the biogeographic history of an Antarctic adaptive radiation

**DOI:** 10.1186/s12862-015-0362-9

**Published:** 2015-06-11

**Authors:** Thomas J Near, Alex Dornburg, Richard C Harrington, Claudio Oliveira, Theodore W Pietsch, Christine E Thacker, Takashi P Satoh, Eri Katayama, Peter C Wainwright, Joseph T Eastman, Jeremy M Beaulieu

**Affiliations:** Department of Ecology and Evolutionary Biology, Yale University, New Haven, CT 06520 USA; Yale Peabody Museum of Natural History, New Haven, CT 06520 USA; Department of Earth Sciences, University of Oxford, South Parks Road, Oxford, OX1 3AN UK; Department Morfologia, Instituto de Biociências, Universidade Estadual Paulista, Botucatu, São Paulo Brazil; School of Aquatic and Fishery Sciences and Burke Museum of Natural History and Culture, University of Washington, Seattle, WA 98105 USA; Research and Collections, Section of Ichthyology, Natural History Museum of Los Angeles County, 900 Exposition Blvd., Los Angeles, CA 90007 USA; National Museum of Nature and Science, Tsukuba City, Ibaraki 305-0005 Japan; Section of Evolution & Ecology, University of California, Davis, CA 95616 USA; Department of Biomedical Sciences, Ohio University, Athens, OH 45701-2979 USA; National Institute for Mathematical and Biological Synthesis, University of Tennessee, 1122 Volunteer Blvd, Ste. 106, Knoxville, TN 37996 USA

**Keywords:** Ancestral range estimation, Weddellian Province, Notothenioidei, Percomorpha

## Abstract

**Background:**

Antarctic notothenioids are an impressive adaptive radiation. While they share recent common ancestry with several species-depauperate lineages that exhibit a relictual distribution in areas peripheral to the Southern Ocean, an understanding of their evolutionary origins and biogeographic history is limited as the sister lineage of notothenioids remains unidentified. The phylogenetic placement of notothenioids among major lineages of perciform fishes, which include sculpins, rockfishes, sticklebacks, eelpouts, scorpionfishes, perches, groupers and soapfishes, remains unresolved. We investigate the phylogenetic position of notothenioids using DNA sequences of 10 protein coding nuclear genes sampled from more than 650 percomorph species. The biogeographic history of notothenioids is reconstructed using a maximum likelihood method that integrates phylogenetic relationships, estimated divergence times, geographic distributions and paleogeographic history.

**Results:**

*Percophis brasiliensis* is resolved, with strong node support, as the notothenioid sister lineage. The species is endemic to the subtropical and temperate Atlantic coast of southern South America. Biogeographic reconstructions imply the initial diversification of notothenioids involved the western portion of the East Gondwanan Weddellian Province. The geographic disjunctions among the major lineages of notothenioids show biogeographic and temporal correspondence with the fragmentation of East Gondwana.

**Conclusions:**

The phylogenetic resolution of *Percophis* requires a change in the classification of percomorph fishes and provides evidence for a western Weddellian origin of notothenioids. The biogeographic reconstruction highlights the importance of the geographic and climatic isolation of Antarctica in driving the radiation of cold-adapted notothenioids.

## Background

The teleost fishes of the Southern Ocean are unlike any other marine fish fauna on Earth because a single clade of closely related species, the notothenioids, dominates the diversity, biomass and abundance [1,2]. The ecological importance of notothenioids is reflected in their role as a key component of Antarctic marine food webs and as the primary targets of fish harvesting in the Southern Ocean [3-7]. In addition, Antarctic notothenioids are one of the most compelling examples of adaptive radiation among ray-finned fishes [1,8]. They show numerous adaptations to polar environmental conditions, including antifreeze glycoproteins (AFGP) [9,10], and interesting patterns of ecological and lineage diversification [10-12]. Despite the attention paid to notothenioids by evolutionary biologists for more than a century [13], and numerous studies investigating the phylogenetic relationships of notothenioids [14-23], the ability to place the diversification of this lineage into the broader context of acanthomorph teleost diversity has been limited because there is still uncertainty regarding the sister lineage of the clade [24].

Since the early 20th Century it has been clear that notothenioids are related to other percomorph teleosts [25,26], but a confident resolution of their sister lineage has remained elusive for more than 100 years [24,27]. Previous phylogenetic hypotheses of notothenioid relationships based on morphology included the Zoarcoidei [28] or elements of the polyphyletic “trachinoids” as candidate sister lineages [22,29-36]. Molecular phylogenetic analyses consistently resolve notothenioids in the recently delimited species-rich percomorph clade Perciformes [sensu 10] that includes Percidae, Bembridae, Platycephalidae, Bembropidae, Gasterosteidae, Zoarcoidae, all of the Scorpaeniformes and the potentially non-monophyletic Serranidae [27,32,37-40]. Support for the specific sister lineage of notothenioids within the Perciformes in molecular phylogenetic analyses has included Percidae [27,32,41], Congiopodidae [42], Trachinidae [43], Bembropidae [37,38], or a clade containing Percidae, *Serranus* and *Bembrops* [44]. Two studies with a broad sampling of percomorph lineages placed notothenioids in Perciformes, but did not provide a strongly supported hypothesis for their sister lineage [39,40]. This lack of resolution regarding the notothenioid sister lineage hinders our understanding of the evolutionary processes that underlie the origination of this Antarctic adaptive radiation.

The utilization of habitats in the subzero waters of the Southern Ocean represents one of the most extreme ecological transitions among teleost fishes [1,9,45]; however, the biogeographic history of notothenioid diversification is poorly understood. In addition to the Antarctic Clade [8], there are three other major notothenioid taxa (Bovichtidae, *Pseudaphritis urvillii* and *Eleginops maclovinus*) that are distributed in areas adjacent to the Southern Ocean including southern South America, the Falkland Islands, Tristan da Cunha, southern Australia and New Zealand [1,46-51]. The phylogenetic relationships of the major notothenioid lineages and their geographic distribution has led to the hypothesis that diversification of the clade was influenced by the breakup of Eastern Gondwana [1,17,22,48], and estimated divergence times of notothenioids using relaxed molecular clocks appear consistent with the timing of Gondwanan fragmentation [8,44,52]. However, the multitude of candidate sister lineages to notothenioids includes clades that span a broad spectrum of geographic distributions that could potentially undermine the East Gondwanan biogeographic hypothesis. For example, if Percidae is the living sister lineage of notothenioids [27,32,41], there will be limited insight into the origin of either clade from historical biogeographic reconstructions because percids exhibit a Holarctic distribution in freshwater habitats that is quite disjunct from the southern hemisphere cold-temperate, sub-Antarctic and Antarctic distribution of notothenioids. On the other hand, if Congiopodidae (racehorses and pigfish) and notothenioids were resolved as sister lineages, as inferred in a previous molecular phylogenetic analysis [42], their shared geographic distribution in the southern hemisphere would potentially strengthen the hypothesis of an East Gondwanan biogeographic pattern.

In this study, we investigate the phylogenetic resolution of notothenioids within the hyper-diverse Percomorpha [8,10]. A DNA sequence dataset of 10 nuclear genes used in several phylogenetic analyses of percomorph fishes [10,40,53,54], including notothenioids [8], is expanded to include every taxon implicated in previous studies as being related to notothenioids. The phylogenetic analyses of this dataset, which includes more than 650 species of Percomorpha, provides a clear and well-supported hypothesis of the sister lineage of notothenioids. Phenotypic traits important in the study of notothenioid phylogeny were examined to determine if there is morphological support for the resolution of the notothenioid sister lineage in our molecular analyses. We time calibrated this new phylogenetic perspective of notothenioid relationships using Bayesian methods and integrated this phylogenetic framework with a likelihood-based model of ancestral area estimation to investigate the biogeographic history that underlies the notothenioid adaptive radiation.

## Results and discussion

### Phylogenetic resolution of the notothenioid sister lineage

**Figure 1:**
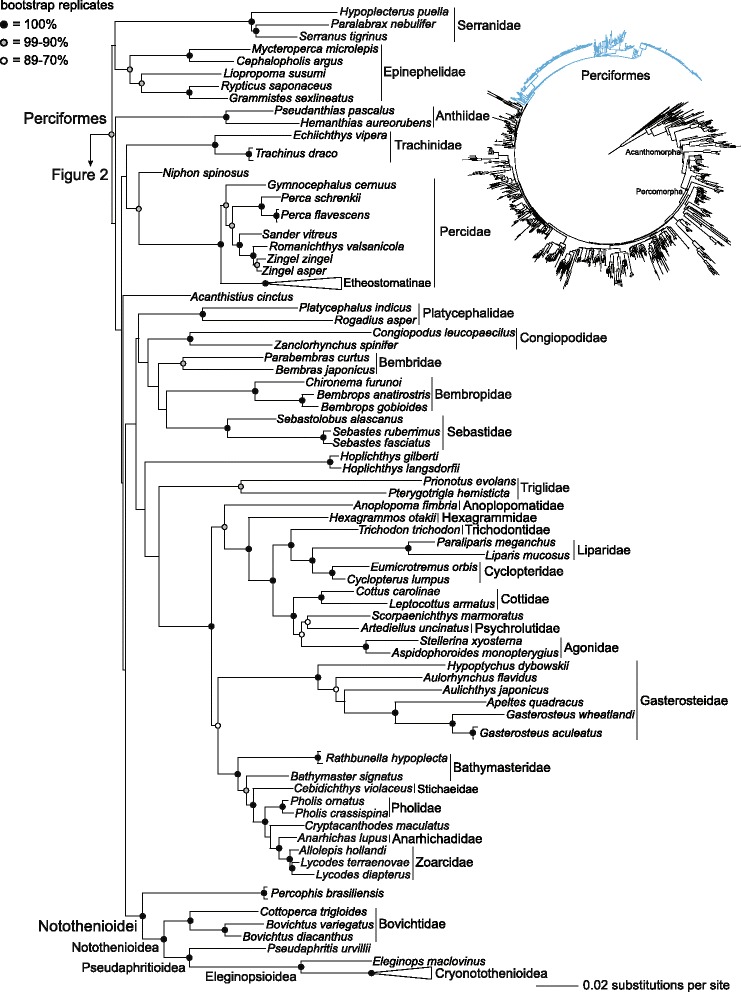
Phylogeny of Perciformes inferred from a partitioned maximum-likelihood analysis of DNA sequences of 10 nuclear genes that resolves *Percophis brasiliensis* as the sister lineage of the Notothenioidea. This is a portion of a larger phylogenetic analysis of acanthomorph teleosts (inset phylogeny with Perciformes highlighted in blue). Filled black circles identify clades supported with a bootstrap score of 100%, filled grey circles identify clades with a bootstrap score between 99% and 90%, and unfilled white circles identify clades with a bootstrap score between 89% and 70%. Polytypic and polygeneric higher-level taxonomic groups are labeled. The clades Acanthomorpha and Percomorpha are identified in the inset tree with filled black circles.

The inferred phylogeny of acanthomorph teleosts resolves relationships among the major notothenioid lineages, namely Bovichtidae, *Pseudaphritis*, *Eleginops* and the Antarctic Clade that are consistent with previous inferences using morphology and molecular data [8,16,17,20,22]. *Percophis brasiliensis* is strongly supported as the sister lineage of notothenioids with a bootstrap score (BSS) of 100 (Figure 1). The expanded notothenioid clade that includes *Percophis* is nested within Perciformes; however, the placement of this lineage among other perciform clades is not well supported (Figure 1). The perciform clades identified as the notothenioid sister lineage in previous molecular phylogenies, Bembropidae, Percidae, Congiopodidae and Trachinidae, are not supported as more closely related to notothenioids than other perciforms in our tree (Figure 1); however, the inferred relationships of these clades relative to the clade containing *Percophis* and the traditional notothenioids are weakly supported (BSS < 70%; Figure 1). The identification of *Percophis* as the sister lineage of all other notothenioids is less a testament to the phylogenetic utility of this particular dataset than simply a result of including the species in the 10 nuclear gene alignment. The only previous DNA sequencing of *Percophis* is the mitochondrial gene COI for barcode studies [55,56].

**Figure 2:**
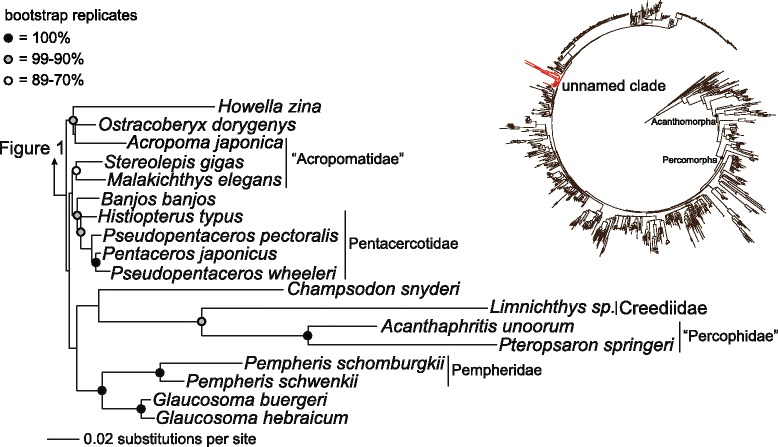
Phylogeny of an unnamed clade of Percomorpha as resulting from an analysis of acanthomorph teleosts (inset phylogeny with the unnamed clade highlighted in red), inferred from a partitioned maximum-likelihood analysis of DNA sequences of 10 nuclear genes. Filled black circles identify clades supported with a bootstrap score of 100%, filled grey circles identify clades with a bootstrap score between 99% and 90%, and unfilled white circles identify clades with a bootstrap score between 89% and 70%. Polytypic and polygeneric higher-level taxonomic groups are labeled. The clades Acanthomorpha and Percomorpha are identified in the inset tree with filled black circles.

The addition of *Percophis*, *Chrionema* and *Pteropsaron* to the molecular phylogenetic dataset demonstrates that the three “percophid” subfamilies resolve in different areas of the percomorph phylogeny (Figures 1 and 2). The paraphyly of Percophidae, which traditionally includes *Percophis,* Bembropidae (*Bembrops* and *Chrionema*) and Hemerocoetinae (*e.g.*, *Acanthaphritis*, *Osopsaron* and *Pteropsaron*) [57-59] is consistent with an earlier phylogenetic analysis of the 10 nuclear gene dataset that sampled *Bembrops* and *Acanthaphritis* and also did not result in monophyly of Percophidae [40]. We resolve *Percophis* and Bembropidae as nested within Perciformes, and Hemerocoetinae, sampled with *Acanthaphritis* and *Pteropsaron*, are resolved as the sister lineage of *Limnichthys* (Creediidae) (Figure 2). A clade containing Hemerocoetinae and Creediidae, as resolved in the molecular phylogeny (Figure 2), was also hypothesized from phylogenetic analysis of 61 morphological characters [60], and is a result that was predicted in other morphological studies that did not rely on optimization of discretely coded character states [33,61-63]. The resolution of notothenioids within “trachinoids” in a previous phylogenetic analysis was potentially the result of morphological synapomorphies shared with *Percophis* [30], but relationships were likely obfuscated by scoring morphological character states for “percophids” as a single taxon that comprised the polyphyletic assemblage comprising *Bembrops*, *Percophis* and *Hemerocoetes* [31,64].

### Morphology and support for a clade containing notothenioids and *Percophis*

Is there morphological evidence to support the molecular phylogenetic analysis that resolves *Percophis* as the sister lineage of notothenioids? Synapomorphies for notothenioids have not been identified and, instead, the lineage is diagnosed by a presumably unique combination of morphological character states: three pectoral radials; poorly developed and floating or absent pleural ribs, especially posteriorly; one nostril on each side of the head; non-pungent fin spines; no swim bladder; two or three lateral lines (occasionally one); jugular pelvic fins; and nasal accessory organs [30,65,66]. While these character states are apomorphic compared to the ancestral percomorph condition [30,33,66-68], they are also homoplastic and occur among various phylogenetically derived percomorph clades and should be regarded with some skepticism given “the rampant homoplasy that has characterized percomorph evolution, particularly at higher levels” ([33] p 22).

Table 1 provides the character states for morphological features used to diagnose notothenioids as a clade [22,36,65,68,69]. We emphasize the character states that differ between the early diverging non-Antarctic notothenioid lineages, Bovichtidae, *Pseudaphritis* and *Eleginops*, with those comprising the Antarctic Clade, and that might be shared with *Percophis*. For three characters, floating pleural ribs, number of nostrils and number of pectoral radials, *Percophis* exhibits the plesiomorphic percomorph state rather than the apomorphic state observed in notothenioids [30,31]. In *Percophis*, posterior pleural ribs articulate with the centra and are not floating. Floating ribs are known in other percomorph lineages including *Trichonotus* [62]. *Percophis* has two nostrils on each side of the head rather than the single nostril characteristic of notothenioids, which is also present in zoarcids and some groups formerly affiliated with zoarcids [29,70]. We note that *Percophis* has a well-ossified skeleton that is similar to that of Bovichtidae and *Pseudaphritis*, which do not display the paedomorphic tendencies toward reduced skeletal ossification and persistence of cartilage that appear in *Eleginops* and all other notothenioids [71].

**Table 1:** Character states for major morphological features of adult *Percophis brasiliensis* and major lineages of Notothenioidea based on radiographs, ethanol preserved and cleared and stained specimens

	**Swim bladder**	**Floating posterior pleural ribs**	**Nostrils on each side of head**	**Pectoral radials**	**Ecto-pterygoid teeth**	**Palatine teeth**	**Ocular choroid rete**	**Persistent choroid fissure**
**Percophidae**								
*Percophis brasiliensis*	−	−	2	4	−	+	+	?^a^
**Bovichtidae**								
*Bovichtus diacanthus*	−	+	1	3	−	+	+	+
*B. variegatus*	−	+	1	3	−	+	+	+
*Cottoperca trigloides*	−	+	1	3	−	+	+	+
*Halaphritis platycephala* ^b^	−	+	1	3	−	+	?	?
**Pseudaphritidae**								
*Pseudaphritis urvillii*	−	+	1^c^	3	+^d^	+	+	−
**Eleginopsidae**								
*Eleginops maclovinus*	−	+	1	3	−	−	+	−
**Nototheniidae**	−	+	1	3	−	−	+ or−	−^e^
**Other families of Cryonotothenioidea**	−	+	1	3	−	−	−or ±	−^e^

Key to symbols and footnotes: +, present; −, absent; ±, vestigial; ?, unknown; ^a^Quality of specimen preservation not sufficient to determine presence/absence; ^b^Data from [48] and A.V. Balushkin (personal communication to J.T. Eastman on the presence of floating posterior pleural ribs in *Halaphritis*); ^c^Larvae of 16.5 mm SL have two nostrils ([76]: p. 64); ^d^In agreement with ([23] p. 43–44), ectopterygoid teeth were present in the three alizarin-stained specimens we examined; ^e^Distal part of choroid fissure persists in *Gobionotothen gibberifrons* and *Dolloidraco longedorsalis* [111]

**Figure 3:**
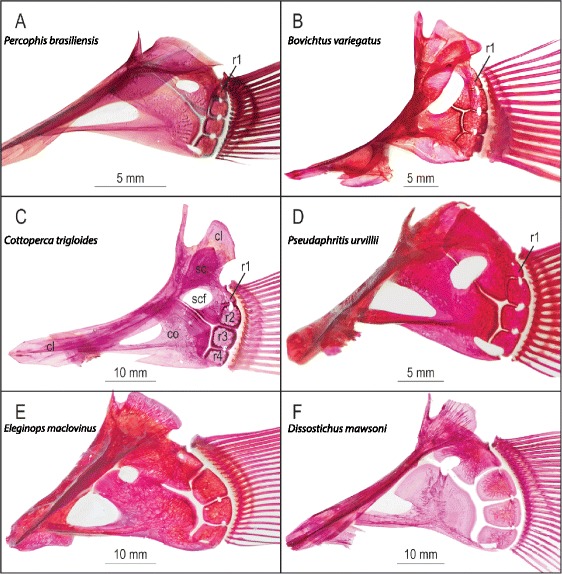
Pectoral girdle morphology in *Percophis brasiliensis* and five species of Notothenioidea. These are left lateral views of alizarin-stained girdles of **(A)** *Percophis brasiliensis* (SL = 115 mm, UW 21233, the specimen illustrated in [31]); **(B)** *Bovichtus variegatus* (SL = 130 mm); **(C)** *Cottoperca trigloides* (SL = 217 mm); **(D)** *Pseudaphritis urvillii* (SL = 180 mm); **(E)** *Eleginops maclovinus* (SL = 260 mm); and **(F)** *Dissostichus mawsoni* (SL = 271 mm). Bones are identified in panel **C** as follows: cl, cleithrum; co, coracoid; r, radials 1–4; sc, scapula; scf, scapular foramen. In *Percophis* **(A)** the dorsal-most radial 1 is relatively small and the suture between it and the scapula is evident in both small **(A)** and large (243 mm SL) specimens [63]. In notothenioids **(B–F)**, radial 1 is present in larvae but, after incorporation into the scapula during development and obliteration of the sutures, it is no longer discrete in adults. The R1 label in **(B–D)** does not indicate the presence of this radial in adults, but rather the approximate location of the anlage of radial 1. *Percophis* **(A)** plus *Bovichtus* **(B)**, *Cottoperca* **(C)** and *Pseudaphritis* **(D)** differ from *Eleginops* **(E)** and *Dissostichus* (Cryonotothenioidea) **(F)** in several respects. In the latter, radials 2–4 are expanded and plate-like **(E & F)**. The maximum anteroposterior length of the pectoral girdle therefore shifts from the posterior margin radial 2 **(A–D)** to the posterior margin of enlarged radial 3 **(E & F)**. This shift changes the articulation pattern among the bones. In *Percophis* **(A)**, *Bovichtus* **(B)**, *Cottoperca* **(C)** and *Pseudaphritis* **(D)**, radial 2 articulates with the scapula whereas in *Eleginops* and Cryonotothenioidea **(E & F)**, it meets both the scapula and the posterior margin of the coracoid [23]. The apparent gaps between individual bones in *Dissostichus mawsoni* **(F)** are filled in life by cartilage. The reduced intensity of the alizarin staining of the coracoid **(F)** of *D. mawsoni* is attributable to the spongy composition of the bone covering the cartilaginous core [71]. In this and other paedomorphic lineages, the pectoral girdle contains considerable persistent cartilage as ossification is delayed and, in some species, is never completed.

**Figure 4:**
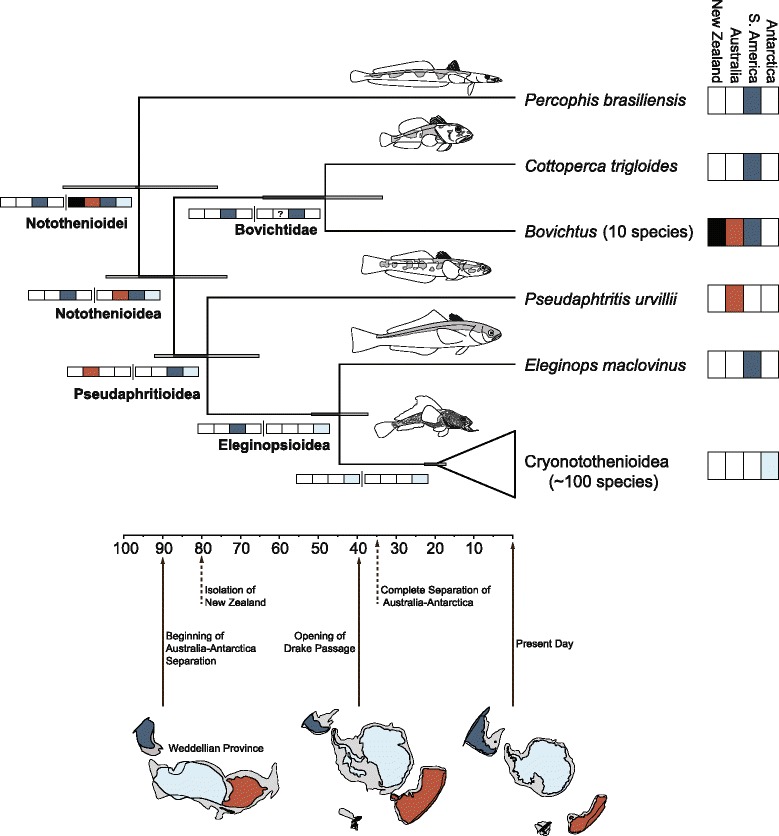
Time-calibrated phylogeny (X-axis in millions of years) and biogeographic reconstructions for the four-area Gondwanan model for Notothenioidei. The constrained maximum-likelihood biogeographic model included four areas corresponding to Gondwanan landmasses. New Zealand (black), Australia, (red), South America (blue) and Antarctica (light blue). The ancestral range shown at each internal node (colored boxes) are the reconstructed scenarios with the highest composite Akaike weight obtain analysis conducted on 1000 randomly chosen phylogenies from the posterior distribution of the Bayesian inferred time trees. The scenarios are drawn to reflect the splitting of the ancestral range due to the speciation event: the colored boxes to the left of the split (black line) represent the range inherited by the upper branch, with the colored boxes to the right of the split represent the range inherited by the lower branch. The timing of major paleogeographic events associated with the fragmentation of the Weddellian Province and East Gondwana are indicated along the x-axis.

Regan ([72] p. 249) first noted the presence of three, *versus* four, radials in the pectoral girdle of notothenioids (Figure 3). There is confusion regarding the number of pectoral radials in *Percophis*, as Boulenger ([73] p. Figure 4 27B) and Pietsch ([31] Figure 2C) both illustrate the *Percophis* pectoral girdle with three radials, and Regan ([74] p. 851) states “the pectoral pterygials number three, one of which is attached to the coracoid in *Ammodytes* and two in *Percophis*”. Our inspection of a cleared and alizarin stained specimen and other accounts in the literature, including Regan ([25] p. 140), show that *Percophis* has four pectoral radials [63]. Although small, the first (dorsal-most) radial in *Percophis* is definitely sutured off from the scapula as a distinct bone (Figure 3A). However, the first radial is less distinctly sutured off than in some other “trachinoid” lineages ([31] p. 260), possibly indicating that it is beginning the process of being ontogenetically lost by incorporation into the scapula. The loss of the first radial is complete in adult notothenioids, and even the non-Antarctic early diverging lineages Bovichtidae, *Pseudaphritis* and *Eleginops,* show no evidence of a suture between the anlage of the first radial and the scapula (Figure 3B–E). All species of notothenioid larvae studied to date have four pectoral radials prior to the fusion of the first radial with the scapula [75-78].

The first pectoral radial has been lost independently in many percomorph lineages [79]. Although still evident, the first radial is also in the process of becoming incorporated into the scapula in some “trachinoid” lineages including pinguipedids, specifically *Pinguipes* ([80] pp. 356–358, Figure 237) and in the creediid *Tewara* [81]. The first pectoral radial is fully incorporated into the scapula in several other percomorph lineages that, like notothenioids, possess only three radials as adults including *Hemerocoetes* ([29] Figure 11b), *Callionymus* ([79] p. 222), and *Bembrops* ([25] p. 141), although some species of *Bembrops* exhibit four radials ([29] p. 49). The recent development of molecular phylogenetic hypotheses for broad sampling of percomorph lineages provides an unusually comprehensive context to examine patterns of morphological evolution in the pectoral skeleton as well as other anatomical systems.

### Percomorph relationships and a new phylogenetic classification of Notothenioidei

Based on the relationships supported in our molecular phylogenetic analysis (Figure 1), we propose several changes to the classification of Percomorpha that are rank-free and based on the principles of phylogenetic nomenclature [82-85]. We propose the name Cryonotothenioidea for the clade informally called the “Antarctic Clade” [8] or the “AFGP-bearing notothenioids” [24,44,52,86], which includes Artedidraconidae, *Harpagifer*, Channichthyidae, Bathydraconidae and Nototheniidae (Figure 1). We provide two additional names: the clade containing *Eleginops* and Cryonotothenioidea is Eleginopsioidea; and the clade containing *Pseudaphritis* and Eleginopsioidea is Pseudaphritioidea (Figure 1). We expand the traditional delimitation of Notothenioidei to include *Percophis* and apply the group name Notothenioidea to the clade containing Bovichtidae, *Pseudaphritis*, *Eleginops* and Cryonotothenioidea (Figure 1). The name Notothenioidea was previously applied to the clade we call Pseudaphritioidea [36].

Some ichthyologists are hesitant to accept taxonomic suggestions based on molecular phylogenetic analyses, preferring morphological evidence for all proposals regarding classification [87]. Since the early days of the 20th century there has been little doubt that Notothenioidea is a natural, or monophyletic, group [13,26,72,88], a hypothesis consistently supported in molecular phylogenetic analyses [32,38,40,41,44]. Despite the confidence in notothenioid monophyly, it is interesting to note that the morphological characters in Table 1 used to define Notothenioidea are homoplastic when considered across the diversity of percomorph teleosts. Hence there is currently no unique character state that either diagnoses notothenioids or that could be used to identify a hypothesized sister lineage, such as *Percophis*, in a revised Notothenioidei (Figure 1). The absence of morphological synapomorphies supporting our new definition of Notothenioidei has no bearing on the merit of the hypothesis that *Percophis* and Notothenioidea share common ancestry, a conclusion supported by our molecular phylogeny and not refuted by morphology. The discovery of synapomorphic character states offering clear support that either *Percophis* or Notothenioidea shares common ancestry with other perciform or percomorph lineages would dispute the monophyly of Notothenioidei, but would still need to be evaluated in the context of other phylogenetic evidence, including molecular data.

### Biogeographic history of notothenioid diversification

The first hypotheses aimed at determining the geographic origin of the Antarctic notothenioids were presented at the beginning of the 20th century [13,26,72,89]. Based on the observations that notothenioids dominate the fish fauna of the Southern Ocean, are relatively species rich and are ecologically and morphologically “peculiar” ([26] p. 40), Regan [26,72] hypothesized that Antarctica and the Southern Ocean were isolated “for a long time, probably throughout the Tertiary Period” ([26] p. 40). Regarding the potential for previous connections between South America, Antarctica and Australia, Regan concluded the distribution of notothenioids “throws no light on the question of former extensions northward of the Antarctic Continent” ([72] p. 2250). The distribution of early diverging non-Antarctic notothenioid lineages, Bovichtidae, *Pseudaphritis* and *Eleginops*, in South America, Australia and New Zealand was explained by dispersal [26,72]. However, this hypothesis was formed in the context of present-day continental configurations before the acceptance of plate tectonic theory.

From the Late Cretaceous through the early Cenozoic, South America, Antarctica Australia and New Zealand were connected in an area of cool temperate shallow seas known as the Weddellian Province [90-93], which has been suggested to comprise the ancestral area of notothenioids ([1] p. 133), [22,69] (Figure 4). Within the Weddellian Province, Balushkin [22] suggested the initial diversification of the notothenioids occurred on the Cretaceous coasts of New Zealand, Australia and Tasmania, a perspective based on the modern-day presence of *Pseudaphritis*, *Halaphtritis*, and *Bovichtus,* and a phylogenetic hypothesis that resolves *Pseudaphritis* as the sister lineage of all other Notothenioidea [22,48].

The combination of Bayesian divergence time information and a likelihood-based method of ancestral range estimation indicates that the formation of the geographic disjunctions observed today among the major notothenioid lineages closely followed the fragmentation of the landmasses encompassing the Weddellian Province (Figure 4, Table 2). This result was built into the DEC model used in our maximum likelihood estimation, given the use of four discrete time intervals to reflect the emergence of South America, New Zealand, Antarctica and Australia as distinct biogeographic regions (see Methods). However, the biogeographic reconstructions are very similar with the removal of these temporal constraints in the DEC analysis, which suggests a strong underlying biogeographic signal among the major notothenioid lineages.

**Table 2:** The three best biogeographic reconstructions for each major notothenioid clade using *lagrange*

**Clade**	**Ancestral Rage**	**AIC Weight (** ***w***_***i***_)	**Evidence Ratio**
Notothenioidei	SA | SA, AU, NZ, AN	0.942	
	*SA | AU, NZ, AN*	0.028	33.21
	*NZ | SA, AU, AN*	0.020	47.28
Notothenioidea	SA | SA, AU, AN	0.467	
	*SA | SA, AN*	0.102	4.59
	*SA | SA, AU*	0.070	6.63
Pseudaphritioidea	AU | SA, AN	0.500	
	*AU | AU*	0.125	3.99
	*AN | SA, AN*	0.083	6.01
Bovichtidae	SA | SA	0.632	
	*SA | SA, AU*	0.130	4.88
	*SA | SA, AU, NZ*	0.101	6.31
Eleginopsioidea	SA | AN	0.637	
	*SA, AU | AN*	0.064	9.90
	*SA | AU, AN*	0.050	12.65
Cryonotothenioidea	AN | AN	0.976	
	*AN | AU, AN*	0.011	88.54
	*AN | SA, AN*	0.009	111.69

The reconstructions used a four-area Gondwanan model that included South America (SA), Australia (AU), New Zealand (NZ), and Antarctica (AN). The optimal ancestral range for each internal node (Figure 4) is listed first and the two less optimal reconstructions are italicized. The scenarios reflect the splitting of the ancestral range with areas to the left of the split represents the range inherited by the upper branch of the phylogeny in Figure 4 and ranges to the right of the split is the range inherited by the lower branch. For each reconstruction the Akaike weight (*w*_*i*_) and evidence ratio are listed.

The first unequivocal biogeographic movements within the notothenioids are associated with the separation of South America from East Gondwana, which, except for the opening of the Drake Passage, was nearly complete by 122 Ma [94] (Figure 4, Table 2). *Percophis* is inferred to have originated in South America, which likely reflects a cladogenic event as the ancestral range expanded to include an isolated South American coastal area, Akaike weight (*w*_i_) = 0.942. The ancestral range of Notothenioidea is inferred as the combination of ranges that comprised the Weddellian Province, and the diversification of the clade involves cladogenesis between Bovichtidae, with an ancestral range of South America, and Pseudaphritioidea in the combined range of Antarctica, Australia and South America (Figure 4, Table 2). The timing of this disjunction, mean age = 88.6 Ma, 95% HPD = 75.0, 106.4 Ma, is very similar to the timing of the initial fragmentation of East Gondwana approximately 90 Ma [95].

The biogeographic origin of the Pseudaphritioidea involves vicariance, specifically the fragmentation of Australia from the remaining East Gondwanan landmasses, *w*_i_ = 0.500 (Figure 4, Table 2). In this scenario, *Pseudaphritis* inherits Australia and the Eleginopsioidea inherit the combined area of Antarctica and South America (Figure 4). The estimated timing of diversification in the Pseudaphritioidea, mean = 80.1 Ma, 95% HPD = 66.6, 93.8 Ma, is close to the initial fragmentation of Australia and Antarctica ~90 Ma [95], but much older than the complete separation of these two landmasses as indicated by the opening of the Tasmanian Seaway ~35 Ma [96].

The timing of diversification in the Eleginopsioidea corresponds closely with the opening of the Drake Passage, which completed the separation of South America and Antarctica (Figure 4). In the most favored biogeographic scenario (*w*_i_ = 0.637), the Cryonotothenioidea remained in Antarctica, while *Eleginops* inherited the South American portion of the ancestral geographic range (Figure 4, Table 2). The Eocene fossil taxon *Proeleginops grandeastmanorum* from Seymour Island, near the Antarctic Peninsula, provides paleontological support for the shared area of South America and Antarctica for Eleginopsioidea, as this taxon is thought to share common ancestry with the South America-Falkland Island endemic *Eleginops maclovinus* [97]. The age of the most recent common ancestor (MRCA) of Eleginopsioidea, mean = 45.6 Ma, HPD = 38.2, 53.0 Ma is similar to the suggested timing (55–41 Ma) of the opening of the Drake Passage, the age of the formation bearing the *P. grandeastmanorum* fossil (52–47 Ma) [98], and the range of estimates for the initial formation of the Antarctic Circumpolar Current (41 to 23 Ma) [99,100].

The estimates of notothenioid range evolution substantiate the previous supposition that the geographic distribution of the major lineages was shaped by the fragmentation of East Gondwana and that the Weddellian Province is the ancestral region of the clade [1,22] (Figure 4, Table 2). Initial diversification of notothenioids is centered in the western portion of the Weddellian Province, particularly South America, involving two instances of vicariance in the MRCA of *Percophis* and the Notothenioidea and the MRCA of Bovichtidae and Pseudaphritioidea (Figure 4). An evaluation of the geographic distribution of Southern Ocean fishes led Andriashev in the 1960s to hypothesize that notothenioids originated in South America ([101] p. 542), but subsequent studies by Balushkin concluded the clade initially diversified in the eastern region of the Weddellian Province [22]. More recently the phylogeny of notothenioids and their geographic distribution was used to argue that the Antarctic continental shelf represents the ancestral area of notothenioids, as interpreted from a so called “center of origin” perspective [102]. Our new analyses synthesize knowledge of the phylogenetic relationships and geographic distribution of notothenioid species with the paleogeography of Eastern Gondwana to discriminate among these alternative biogeographic scenarios and provide the strongest support that the western Weddellian Province, centered on South America, was the area of initial diversification for the clade.

## Conclusions

Phylogenetic analysis of DNA sequences sampled from 10 exon regions across a wide diversity of percomorph teleosts provides strong support for *Percophis brasiliensis* as the sister lineage of all other notothenioids (Figures 1 and 4). This result solves a century-old evolutionary puzzle, as the first scientists to describe the fish fauna of the Southern Ocean were unsure as to the relationships of notothenioids among the major lineages of percomorph teleosts [13,26,72,89]. The resolution of *Percophis* as the sister lineage of all other notothenioids is used to change the classification of percomorph fishes and contributes to the strong inference that southern South America, as associated with the western portion of the East Gondwana Weddellian Province, as the ancestral area of notothenioid diversification (Figure 4). The biogeographic history of notothenioid diversification estimated in our study illuminates the temporal and spatial circumstances that resulted in an interesting contrast between the species-depauperate relictual lineages *Percophis*, Bovichtidae, *Pseudaphritis* and *Eleginops*, with the eventual physical and climatic isolation of the Southern Ocean and the subsequent adaptive radiation of the species-rich Cryonotothenioidea.

## Methods

### Taxonomic sampling, DNA sequencing and phylogenetic analysis

The phylogenetic analyses in this study utilize DNA sequences of 10 nuclear protein coding genes sampled from all 550 species of Acanthomorpha included in Near *et al*. [40], expanded here to include 738 species. The taxon sampling includes 83 notothenioids [8], 101 species of Percidae [53] that includes *Perca schrenkii*, *Gynocephalus cernuus*, *Romanichthys valsanicola* and *Zingel asper*, which were sampled for this study, two species of the non-monophyletic Serranidae (*Acanthistius cinctus* and *Liopropoma susumi*) [37, 40], two species of Trachinidae (*Trachinus draco* and *Echiichthys vipera*), two species of Congiopodidae (*Congiopodus leucopaecilus* and *Zanclorhynchus spinifer*), a species of Bembropidae (*Chrionema furunoi*), and two species of Percophidae (*Pteropsaron springeri* and *Percophis brasiliensis*). The addition of these newly sampled species to the taxon sampling in the Near *et al.* [40] dataset ensures that every lineage identified as the sister lineage of notothenioids is sampled and there is a dense sampling of lineages that comprise the Perciformes [sensu 10]. All field collection and processing of specimens followed the American Society of Ichthyologists and Herpetologists Guidelines for the Use of Fishes in Research (http://www.asih.org/publications).

Qiagen DNeasy Blood and Tissue kits were used to isolate DNA from tissue biopsies. Using isolated genomic DNA as a template previously published PCR primers [103, 104] were used to amplify and sequence a single exon from each of ten unlinked nuclear encoded genes *ENC1, Glyt, myh6, plagl2, Ptr, rag1, SH3PX3, sreb2, tbr1* and *zic1*. These 10 protein coding gene regions were aligned by eye to the dataset used in Near *et al.* [40] and confirmed by examination of alignments of the inferred amino acid sequences. No frame mutations or DNA substitutions that resulted in stop codons were observed in the aligned sequences. The combined ten-gene dataset contained 8,577 base pairs. The outgroup taxa were the same set of seven sampled ostariophysan species used in Near *et al.* [40].

Thirty data partitions were designated that corresponded to the three separate codon positions for each of the ten protein coding genes. A GTR + Γ_4_ substitution model was used in a partitioned maximum likelihood analysis using the computer program RAxML 7.2.6 [105], run with the –D option, and 500 maximum likelihood searches. Support for nodes in the RAxML tree was assessed with a thorough bootstrap analysis (option –f i) with 500 replicates.

### Molecular divergence time estimates

Relaxed molecular clock methods were used to estimate divergence times among major lineages of notothenioids and the sister lineage of the clade. Divergence time analyses were performed on a subset of seven species that included *Percophis brasiliensis*, which is resolved as the sister lineage of Notothenioidei, two species of Bovichtidae (*Bovichtus diacanthus* and *Cottoperca trigloides*), *Pseudaphritis urvillii* (the only species classified in Pseudaphritidae), *Eleginops maclovinus* (the only species classified in Eleginopsidae), and two species sampled to include the MRCA of Cryonotothenioidea (*Dissostichus eleginoides* and *Chionobathyscus dewitti*). Divergence times were estimated using the uncorrelated lognormal (UCLN) model of molecular evolutionary rate heterogeneity implemented in the computer program BEAST v. 1.8 [106,107]. The ten-gene dataset was partitioned as in the maximum likelihood RAxML phylogenetic analysis, unlinking the nucleotide substitution models among the 30 codon-based partitions and the UCLN clock model was partitioned among the 10 genes.

Based on the results of a previous UCLN analyses [10,44], age priors with a normal distribution were applied to three nodes in the notothenioid phylogeny, which included the MRCA of *Pseudaphritis urvillii* and all other Eleginopsioidea (mean = 63.0, standard deviation = 10.4), the MRCA of *Eleginops maclovinus* and the Antarctic clade (mean = 42.9, standard deviation = 8.0), and the MRCA of the Antarctic clade (mean = 23.8, standard deviation = 1.5). The fossil notothenioid *Proeleginops grandeastmanorum* was not used as a calibration, as it would be the only fossil-based age prior [97]. However, the age priors used are the result of relaxed molecular clock analyses that broadly sampled the lineage diversity of acanthomorph and percomorph teleosts and used multiple non-notothenioid fossil calibrations [40, 44]. A birth-death speciation prior was used for branching rates in the phylogeny. The BEAST analyses were run five times with each run consisting of 3.0 × 10^8^ generations, sampling at every 10,000 generations. The resulting trees and log files from each of the five runs were combined using the computer program LogCombiner v. 1.8 (http://beast.bio.ed.ac.uk/LogCombiner). Convergence of model parameter values and estimated node-heights to their optimal posterior distributions was assessed by plotting the marginal posterior probabilities versus the generation state in the computer program Tracer v. 1.5 (http://beast.bio.ed.ac.uk/Tracer). Effective sample size (ESS) values were calculated for each parameter to ensure adequate mixing of the MCMC (ESS > 200). The posterior probability density of the combined tree and log files was summarized as a maximum clade credibility tree using TreeAnnotator v. 1.8 (http://beast.bio.ed.ac.uk/TreeAnnotator).

### Estimating the biogeographic history of notothenioids

The dispersal-extinction-cladogenesis model (DEC) was used to estimate the biogeographic history for the major notothenioid lineages [108]. The DEC model assumes dispersal-mediated range expansion and extinction-mediated range contraction, with the probability of either event occurring along a particular branch being proportional to the length of that branch and the transition rates between geographic areas [108]. The transition rates and the reconstruction of the likeliest dispersal scenarios at all internal nodes under the DEC model were estimated using the C++ version of *lagrange*.

We altered the migration probabilities among the four biogeographic regions in our model—Antarctica, South America, Australia or New Zealand—to reflect changes in connections to these areas during the gradual fragmentation of Gondwana. This involved devising separate migration matrices for four discrete time intervals: 150–80 Mya, 80–50 Mya, 50–30 Mya and 30–0 Mya. For the 150–80 Mya interval we assumed a zero probability of any movement our defined areas for the time period leading up to 80 Myr. This ensured that lineages were not estimated as being in Antarctica, South America, Australia or New Zealand in isolation when none of these regions technically existed. Non-zero probabilities for movement were allowed in the time interval of 80–50 Myr to New Zealand and South America to reflect the isolation of the landmasses from the combined region of Australia-Antarctica during this time period. From 50 to 30 Myr, the possibility of successful movements to Australia were allowed, as this was the time period in which it completed its separation from Antarctica. Finally, the 30–0 Mya interval reflected the emergence of the current geographic configuration of Antarctica, South America, Australia and New Zealand. The sensitivity of the reconstructions to the temporal constraints was examined by running the *lagrange* analysis with these constraints relaxed.

In order to assess the impact of both phylogenetic and temporal uncertainty on the ancestral range estimates, we inferred the most likely biogeographic scenarios across 1,000 randomly chosen trees obtained from the posterior distribution of time trees inferred using BEAST. We relied on a composite Akaike weights (*w*_i_) as a means of summarizing biogeographic estimates of ancestral ranges across the posterior set of trees [109]. The composite Akaike weight (*w*_i_) for a given scenario is the average of the individual Akaike weights calculated for each tree separately. Thus, we interpret the composite Akaike weight as describing the average relative likelihood of a given biogeographic scenario over a set of all possible alternative scenarios [109]. As *lagrange* only reports ancestral area estimates that are less than two-log likelihood units away from the inferred global likelihood, we utilized a modified version of *lagrange* that outputs the likelihood of all possible biogeographic scenarios estimated at a focal node [109] that has also been used in other biogeographic studies [110].

## Availability of supporting data

The data sets supporting the results of this article are available in the Dryad repository, [doi:http://dx.doi.org/10.5061/dryad.s355k]. All new DNA sequences are submitted to Genbank (KP965919-KP966072).
